# The inflammatory clock: how cGAS-STING ticks in the aging ovary

**DOI:** 10.3389/fcell.2026.1771546

**Published:** 2026-02-24

**Authors:** Yanjing Ma, Yu Chen, Xiong Yuan, Tingyu Li, Hao Luo, Yanfang Gu

**Affiliations:** 1 Department of Gynecology, Wuxi Maternal and Child Health Hospital, Wuxi School of Medicine, Jiangnan University, Wuxi, Jiangsu, China; 2 Department of Gynecology, Wuxi Maternal and Child Health Hospital, Wuxi, Jiangsu, China

**Keywords:** cGAS-STING, inflammation, ovarian aging, premature ovarian insufficiency, therapeutic targets

## Abstract

Premature ovarian insufficiency (POI) is more than a fertility issue; it's a silent epidemic of accelerated systemic aging in young women, with current treatments failing to address its root cause. For too long, the relentless decline of ovarian function has been viewed as an inevitable mystery. But what if the ovary holds an internal “inflammatory clock,” ticking away with each cellular insult and dictating the pace of its own decline? Here, we spotlight a surprising culprit: the cGAS-STING signaling pathway. Far beyond its day job in antiviral defense, this pathway emerges as a master integrator of ovarian aging. We reveal how stresses like DNA damage and mitochondrial dysfunction leak genetic material into the cell’s interior, where cGAS-STING sounds a relentless alarm. This alarm does not just trigger inflammation; it initiates a vicious, self-amplifying cycle of cellular senescence, tissue fibrosis, and follicle destruction—a cycle that may explain why ovarian aging often feels like a one-way street. Therapeutically, we move beyond mere symptom management to explore strategies for resetting this inflammatory clock. We dissect both direct “brakes”—novel small molecules that silence cGAS or STING—and upstream “shields” that protect cellular powerplants and genome integrity. Most provocatively, we introduce the concept of “signal reprogramming”: not just shutting down the pathway, but cleverly rewiring its output to favor repair over destruction. By repositioning cGAS-STING from a simple sensor to the central processor of ovarian aging, this review charts a course for a new class of therapeutics aimed at preserving ovarian function, not just managing its loss. The goal is no less than transforming our approach to women’s reproductive longevity.

## 1 Introduction

Ovarian aging is one of the earliest and most rapidly occurring forms of organ aging in the female reproductive system, often beginning in early adulthood (Te Velde and Pearson, 2002; Zhu et al., 2021). This process culminates clinically in premature ovarian insufficiency (POI), a condition of accelerated ovarian aging characterized by hypergonadotropic hypogonadism before age 40 (Touraine et al., 2024; Roeters van Lennep et al., 2016). Recent data suggest that the prevalence of POI is higher than previously estimated, affecting 3.5%–3.7% of women (Golezar et al., 2019; Li et al., 2023). This indicates that POI is not a rare condition. Beyond impairing fertility and reducing assisted reproductive success, it is associated with early-onset atherosclerosis, osteoporosis and low-energy fractures, metabolic syndrome, type 2 diabetes, increased risk of Alzheimer’s disease, as well as depression and anxiety (Roeters van Lennep et al., 2016; Gatenby and Simpson, 2024), displaying characteristics of a systemic chronic disease with public health implications that extend far beyond reproductive outcomes (Panay et al., 2024).

Current management relies on hormone replacement therapy (HRT) to alleviate symptoms and mitigate some long-term risks (Costa-Paiva et al., 2022; Nie et al., 2022). However, HRT fails to restore the ovarian follicle reserve or reverse the underlying degenerative process. A critical challenge is the protracted subclinical phase of POI, where significant ovarian damage often precedes diagnosis by years (Jiao et al., 2021). This clinical irreversibility suggests the existence of self-perpetuating molecular pathways that lock the ovary into a degenerative trajectory.

Ovarian aging is driven by a complex, interconnected network of mechanisms: genomic instability, mitochondrial dysfunction, telomere attrition, and a persistent state of sterile inflammation (Wu et al., 2025; Hua et al., 2020; May-Panloup et al., 2005; Kosebent et al., 2021). Emerging as a central integrator of these diverse stressors is the cyclic GMP–AMP synthase–stimulator of interferon genes cGAS–STING pathway (Dvorkin et al., 2024). Beyond its canonical role in antiviral defense, cGAS-STING acts as a cytosolic DNA sensor, orchestrating a profound inflammatory and senescent response upon detection of misplaced self-DNA (Barber, 2015; Gao et al., 2013). Accumulating evidence indicates that components of the cGAS–STING pathway exhibit age- and disease-associated alterations in the ovary, including increased expression of cGAS and STING, enhanced STING phosphorylation, and sustained downstream activation of type I interferon and NF-κB signaling in aging ovarian tissue and experimental models of POI (Cabral et al., 2021; Cheng et al., 2025; Pan et al., 2025). These progressive and persistent activation patterns suggest that cGAS–STING signaling may act as an “inflammatory clock,” translating cumulative cellular damage into a feed-forward loop of chronic inflammation that accelerates ovarian functional decline. In this review, we systematically delineates the mechanistic evidence for this pathway’s role and explores its potential as a novelcyclic GMP–AMP synthase–stimulator of interferon genes cGAS–STING therapeutic target from the perspective of re-establishing dynamic pathway homeostasis. This review systematically delineates the mechanistic evidence for this pathway’s role and explores its potential as a novel therapeutic target from the perspective of re-establishing dynamic pathway homeostasis.

## 2 The cGAS-STING signaling axis: from cytosolic DNA sensing to sterile inflammation

**FIGURE 1:**
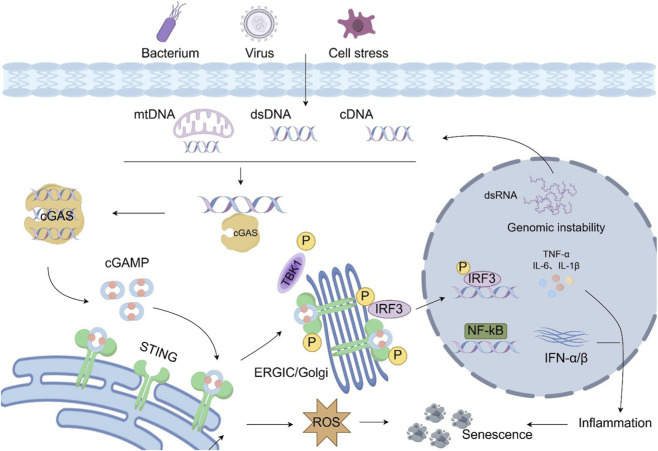
Molecular mechanism of cGAS-STING signalling. The initial function of cGAS-STING signalling is to defend host cells against pathogens, and later studies have shown that this pathway is also related to inflammatory diseases and senescence. Upon sensing cytosolic DNA, cGAS becomes activated and catalyzes the synthesis of the second messenger cGAMP, which subsequently activates STING located on the endoplasmic reticulum. Activated STING translocates to the Golgi apparatus and recruits TBK1 kinase, leading to the phosphorylation and nuclear translocation of the downstream transcription factor IRF3, thereby inducing type I interferon expression. Meanwhile, STING can also activate the NF-κB signaling pathway through the IKK complex, promoting the production of various pro-inflammatory cytokines and thereby jointly mediating the onset and progression of inflammation and cellular senescence. cGAMP, cyclic GMP-AMP; cGAS, cyclic GMP-AMP synthase; ER,endoplasmic reticulum; ERGIC, ER–Golgi intermediate compartment; IFN, interferon; IRF-3, interferon regulatory factor-3; STING, stimulator of interferon genes; TBK1, TANK-binding kinase 1; ROS, reactive oxygen species; IL-6,Interleukin-6; IL-1β,Interleukin-1β; TNF-α, tumor necrosis factor-α.

The cGAS–STING pathway signaling pathway serves as a central hub for sensing cytosolic DNA and initiating innate immune and senescence responses, extending beyond its classical role in antiviral defense (Sun et al., 2013). As shown in Figure 1, under homeostatic conditions, nuclear DNA and mitochondrial DNA (mtDNA) are strictly compartmentalized within double-membrane systems, maintaining spatial fidelity of genetic information and cellular homeostasis. Notably, cGAS is also present in the nucleus; however, its ability to recognize self-DNA is actively suppressed through multiple regulatory mechanisms. One key mechanism involves nucleosome tethering, whereby cGAS is bound to nucleosomes in a conformation that prevents spurious activation by genomic DNA, thus maintaining tolerance to self-DNA and preventing unwarranted inflammatory signaling (Boyer et al., 2020; Volkman et al., 2019)^.^ Other nuclear retention and inhibitory mechanisms further restrict cGAS activation, ensuring that nuclear DNA does not aberrantly trigger the cGAS–STING pathway under physiological conditions. However, upon DNA virus infection, telomere attrition, DNA replication stress, oxidative stress, or aging-associated damage, nuclear or mitochondrial DNA can partially leak into the cytosol, where it is sensed by cGAS (cyclic GMP–AMP synthase) (Luecke et al., 2017; Ablasser et al., 2013). These regulatory mechanisms have primarily been validated in non-ovarian cell models or *in vitro* experiments, and their universality and specificity in ovarian cells, including granulosa cells and oocytes, remain critical questions for future investigation.

cGAS is a highly conserved double-stranded DNA (dsDNA) sensor that is activated upon dimerization induced by dsDNA binding, catalyzing the synthesis of the second messenger 2′3′-cGAMP from GTP and ATP (Zhang et al., 2025). As a signaling mediator, cGAMP binds to STING (stimulator of interferon genes), inducing STING oligomerization and conformational changes, leading to its translocation from the endoplasmic reticulum (ER) to the ER–Golgi intermediate compartment (ERGIC/Golgi), thereby providing a platform for downstream signaling (Gao et al., 2013). STING functions not only as a receptor for cGAMP but also as a signaling integrator; its conformational change enables the recruitment of multiple downstream kinases and transcription factors, converting cytosolic DNA sensing into broad immune and senescence signaling (Ablasser et al., 2013; Diner et al., 2013).

TBK1 (TANK-binding kinase 1) is a key bridging kinase that phosphorylates both STING and interferon regulatory factor 3 (IRF3), promoting IRF3 dimerization and nuclear translocation to initiate transcription of type I interferons (IFN-α/β) and interferon-stimulated genes (ISGs). These ISGs play central roles in antiviral defense and immune regulation while also contributing to cellular stress responses and DNA repair (Wu et al., 2013; Harding et al., 2017). Concurrently, NF-κB signaling is activated, inducing the expression of pro-inflammatory cytokines such as TNF-α, IL-6, and IL-1β, generating an inflammatory cascade and exerting paracrine effects on neighboring cells and the tissue microenvironment through SASP factors (Mackenzie et al., 2017). SASP factors not only amplify local inflammation but also induce cellular senescence and bystander effects, creating a chronic low-grade inflammatory milieu that plays a central pathological role in tissue degeneration and organ functional decline (Glück et al., 2017).

Beyond classical inflammatory pathways, STING participates in autophagosome formation and mitochondrial homeostasis, providing negative feedback for damaged DNA clearance and impaired mitochondrial regulation (West et al., 2015). However, under chronic or sustained activation, this negative feedback may become dysregulated, resulting in reactive oxygen species (ROS) accumulation that exacerbates DNA damage, forming a bidirectional cGAS–STING–ROS positive feedback loop (McArthur et al., 2018; Motwani et al., 2019; Hopfner and Hornung, 2020). This loop not only amplifies cellular senescence signals but may also accelerate tissue-level degeneration (Yang et al., 2017a). Particularly in the ovary, chronic activation of this pathway may directly drive oocyte apoptosis, granulosa cell senescence, and deterioration of the follicular microenvironment, representing a central mechanism in POI onset and rapid ovarian functional decline. Nonetheless, this theoretical model is largely extrapolated from other tissues or disease models, and its precise contribution to ovarian aging, as well as the presence of ovary-specific regulatory factors, still requires direct experimental validation.

## 3 cGAS-STING as a hub integrating multiple drivers of ovarian aging

Ovarian aging involves multiple layers of molecular and cellular mechanisms, including the progressive accumulation of nuclear and mitochondrial DNA damage, impaired DNA repair, chromosomal abnormalities, telomere attrition, mitochondrial dysfunction, and sustained elevation of ROS in oocytes and ovarian somatic cells (May-Panloup et al., 2005; Qin et al., 2025; Titus et al., 2013). Available evidence suggests that these age-associated stressors do not act independently but tend to converge, establishing a persistent state of low-grade sterile inflammation, characterized by increased production of SASP factors, which potentially remodel the ovarian microenvironment and compromise follicle development and reserve.

Importantly, ovarian aging is intrinsically a multicellular process. Distinct ovarian cell populations—including oocytes, granulosa cells, theca cells, stromal fibroblasts, and resident immune cells—appear to exhibit differential sensitivities to genomic stress, oxidative damage, and inflammatory cues. Granulosa cells, for example, appear particularly susceptible to DNA damage and ROS, readily displaying senescence markers with functional decline (Titus et al., 2013; Zhang et al., 2015). Stromal cells, by contrast, can mediate paracrine signaling through SASP factor secretion, amplifying local inflammatory responses (Glück et al., 2017; Shen et al., 2023). Accumulating evidence indicates that specific stressors, such as persistent DNA lesions, oxidative stress, and mitochondrial dysfunction, are closely associated with the development of primary ovarian insufficiency (POI) (Hong et al., 2025; Han et al., 2025). These findings support the notion that POI is a multi-cell type disease, in which coordinated dysfunction of heterogeneous ovarian cell populations underlies its pathogenesis. Furthermore, certain subsets of cells—particularly granulosa cells experiencing high replication stress or oocytes harboring accumulated DNA damage—exhibit more robust activation of the cGAS–STING pathway, thereby amplifying senescence-associated inflammatory signaling and accelerating ovarian aging (Yan et al., 2024; Zhou et al., 2024).

Notably, the heterogeneous stress responses observed among ovarian cell types are further shaped by ovary-specific biological features that fundamentally distinguish the ovary from most classical somatic tissues. Oocytes remain arrested at the diplotene stage of meiosis I for prolonged periods—often spanning decades in humans—during which they must maintain genome integrity in the absence of cell division (Titus et al., 2013). This prolonged meiotic arrest imposes unique constraints on DNA damage sensing, repair pathway selection, and tolerance to persistent lesions (Gruhn et al., 2019). In contrast, granulosa cells undergo rapid and repetitive proliferation–differentiation cycles during folliculogenesis, rendering them particularly vulnerable to replication-associated DNA damage and oxidative stress (Zhang et al., 2015).

Together, these intrinsic ovarian features create a cellular environment characterized by an elevated burden of genomic instability and mitochondrial stress, potentially increasing the likelihood of cytosolic DNA accumulation across multiple ovarian cell types. Within this multi-layered aging context, the cyclic GMP–AMP synthase–stimulator of interferon genes (cGAS–STING) pathway emerges conceptually as a central signaling hub that links upstream DNA damage and mitochondrial dysfunction with downstream inflammatory responses, cellular senescence, and ROS amplification. Age-associated increases in DNA damage burden and mitochondrial dysfunction are therefore expected to progressively lower the activation threshold of cGAS–STING signaling in multiple ovarian cell types, reinforcing chronic inflammation and senescent remodeling of the ovarian niche. In this framework, cGAS–STING may function as an “inflammatory clock” of the ovary.

**FIGURE 2:**
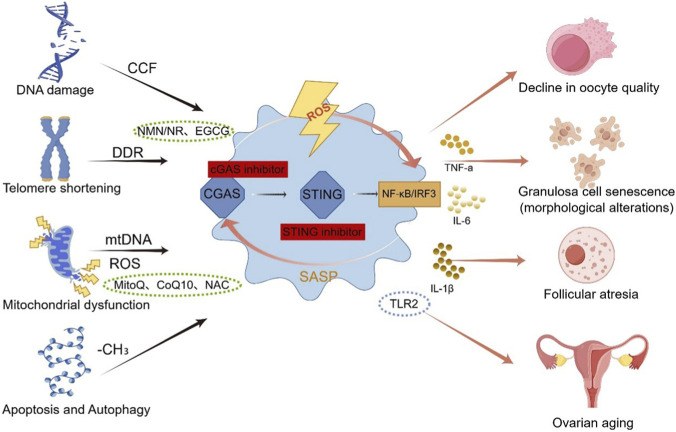
cGAS–STING pathway in ovarian aging and potential intervention targets. During ovarian aging, DNA damage, telomere shortening, and mitochondrial dysfunction lead to cytoplasmic DNA leakage, including micronuclei and mitochondrial DNA (mtDNA), which activates the DNA sensor cGAS. Activated cGAS catalyzes the synthesis of the second messenger cGAMP, subsequently activating STING. STING recruits TBK1 kinase, inducing phosphorylation of NF-κB and IRF3 and promoting expression of pro-inflammatory cytokines such as TNF-α, IL-6, and IL-1β, forming a senescence-associated secretory phenotype (SASP). These inflammatory factors impair oocyte quality, accelerate granulosa cell senescence, and promote follicular atresia, accelerating ovarian functional decline. In the figure, red boxes indicate direct cGAS/STING inhibitors; green dashed boxes indicate upstream drugs (MitoQ, CoQ10, NAC, NMN/NR, EGCG) that indirectly suppress cGAS–STING by reducing oxidative stress and enhancing DNA repair; blue dashed boxes indicate TLR2 agonists, which precisely modulate downstream STING signaling to “reprogram” immune output and reduce chronic inflammation. cGAMP, cyclic GMP–AMP; cGAS, cyclic GMP–AMP synthase; STING, stimulator of interferon genes; TBK1, TANK-binding kinase 1; NF-κB, nuclear factor κB; IRF3, interferon regulatory factor 3; ROS, reactive oxygen species; mtDNA, mitochondrial DNA; SASP, senescence-associated secretory phenotype; TNF-α, tumor necrosis factor α; IL-6, interleukin 6; IL-1β, interleukin 1β; NAC, N-acetylcysteine; NMN, nicotinamide mononucleotide; NR, nicotinamide riboside; EGCG, epigallocatechin-3-gallate; CoQ10, coenzyme Q10; TLR2 agonist, Toll-like receptor 2 agonist.

A key unresolved question, however, is whether distinct ovarian cell populations—such as oocytes and granulosa cells—exhibit differential activation thresholds, signaling outputs, or functional consequences of cGAS–STING signaling, and future studies in cell type–specific ovarian models are needed to address this question In Figure 2.

### 3.1 Nuclear DNA damage

Nuclear DNA damage and the subsequent leakage of genetic material into the cytosol are considered a critical initiating events in cellular senescence and tissue aging. Genotoxic insults from chemotherapy, environmental toxins, or the natural aging process induce DNA double-strand breaks, genomic instability, and telomere shortening across multiple ovarian cell types, including granulosa cells, oocytes, and stromal cells, with oocytes and granulosa cells being particularly vulnerable because of their limited DNA repair capacity and high metabolic demand. Importantly, the age-associated accumulation of DNA damage in granulosa cells may reflect both a consequence of aging-related decline in repair mechanisms and a driving force that promotes further cellular senescence and ovarian functional decline. In oocytes, this vulnerability is further compounded by their reliance on long-term DNA damage tolerance rather than continuous cell cycle–coupled repair, a feature that distinguishes germ cells from proliferating somatic cells and predispose them to persistent cytosolic DNA signaling when repair capacity is exceeded (Stringer et al., 2020).

Such damage can lead to the formation of cytoplasmic chromatin fragments (CCFs), which serve as potent ligands for cGAS recognition and pathway activation (Dou et al., 2017; Shen et al., 2021). Mechanistic insights into CCF-mediated cGAS activation are primarily derived from studies in senescent fibroblasts and tumor cells, while direct experimental validation in ovarian cells remains limited. Nevertheless, emerging observations in aging ovarian tissues suggest that analogous processes may occur under conditions of sustained genomic stress., and it is plausible that age-dependent cytosolic DNA accumulation in granulosa cells could engage cGAS–STING signaling, amplifying downstream NF-κB and IRF3 responses. Recent studies indicate that the integrity of the nuclear envelope is a key regulator of this process; for example, downregulation of the nuclear lamina protein Lamin B1 has been shown in non-ovarian models to accelerates nuclear envelope rupture and CCF formation. Concurrently, impaired degradation of cytosolic DNA, such as through deficient TREX1 enzyme activity, results in persistent cGAS stimulation (Frediani et al., 2022; Mohr et al., 2021).

Upon binding to cytosolic DNA, cGAS catalyzes the synthesis of 2′3′-cGAMP, which activates STING and downstream NF-κB and IRF3 signaling axes. NF-κB activation induce the expression of pro-inflammatory cytokines (e.g., IL-6, TNF-α) and can also influence genomic stability by modulating the activity of DNA repair factors, such as those involved in homologous recombination (Xu et al., 2024). Within the ovarian context, NF-κB functions not only as an amplifier of DNA damage–induced stress signaling but also indirectly affects oocyte maturation and follicular microenvironment homeostasis through the regulation of pro-inflammatory and pro-fibrotic factors (Sze et al., 2022; García-García et al., 2021). The link between DNA damage and NF-κB activation is particularly evident in granulosa cells. Studies comparing granulosa cells from young and aged mice have revealed an age-dependent increase in the DNA damage marker γH2AX, which correlates with enhanced NF-κB nuclear localization, suggesting that DNA double-strand breaks are potent activators of NF-κB signaling in these cells (Zhang et al., 2015). This age-dependent DNA damage may both result from diminished DNA repair capacity during aging and act as a driver of granulosa cell senescence, potentially through activation of the cGAS–STING pathway. Supporting this notion, *in vitro* experiments demonstrate that inhibition of NF-κB transcriptional activity attenuate DNA damage–induced expression of pro-inflammatory cytokines and partially rescue cell proliferation, positioning NF-κB as a critical signal amplifier in granulosa cell senescence. Furthermore, in models of age-related ovarian fibrosis, aberrant NF-κB activation in stromal cells is associated with the accumulation of pro-fibrotic factors such as TGF-β1, and inhibition of NF-κB alleviates this fibrotic response (Grzeczka et al., 2025). This cascade can be further exacerbated by the activation of PARP1 in response to DNA damage, which depletes intracellular NAD^+^ pools and promotes cellular senescence, thereby forming a reinforcing loop with NF-κB signaling and senescence-associated secretory phenotype (SASP) factors (Salminen et al., 2012).

In parallel, STING activation recruits TBK1 to phosphorylate IRF3, initiating the transcription of type I interferons (IFN-α/β) and interferon-stimulated genes (ISGs) (Yu and Liu, 2024). In the early stages of DNA damage, this interferon response may exert context-dependent protective effects, including enhancement of cellular defense mechanisms and maintenance of mitochondrial homeostasis (Li and Chen, 2018). However, under conditions of chronic or irreparable genomic damage, persistent IRF3/type I interferon signaling is hypothesized to disrupt ovarian cellular homeostasis, thereby accelerating follicle depletion and oocyte quality decline (Titus et al., 2013; Yu et al., 2015; Mao et al., 2024), Clinical observations of elevated DNA damage markers, inflammatory cytokines, and type I interferon signatures in aged ovaries are consistent with the potential involvement of the cGAS–STING–NF-κB/IRF3 axis in human ovarian aging, although direct causal evidence in ovarian cells is still limited (Al Hamrashdi and Brady, 2022; Cao, 2022; Mao et al., 2024).

In summary, nuclear DNA damage in the ovary initiates a deleterious signaling cascade—DNA damage/CCF formation/cGAS–STING activation/NF-κB and type I interferon signaling/SASP secretion—ultimately driving cellular senescence and functional decline. The type I interferon response thus represents a double-edged sword, and the threshold at which its role shifts from protective to pathological remains a critical area for future investigation.

### 3.2 Mitochondrial DNA (mtDNA) leakage and ROS elevation

Mitochondria play a central role in ovarian aging, functioning both as the primary source of reactive oxygen species (ROS) and as a highly vulnerable target of age-related damage (Yan et al., 2022). ROS—including superoxide anion (O_2_^−^), hydrogen peroxide (H_2_O_2_), and hydroxyl radicals (·OH)—can exert context-dependent effects: under physiological conditions, they participate in essential signaling and cellular defense, whereas persistent or excessive ROS accumulation, resulting from overproduction or impaired clearance, induces oxidative stress and damages proteins, lipids, and nucleic acids (Zhao et al., 2019). Within mitochondria, ROS are inevitable byproducts of oxidative phosphorylation, and the integrity of the mitochondrial membrane is therefore critical for maintaining redox homeostasis, particularly in metabolically active ovarian cells (Zhao et al., 2019; Tirichen et al., 2021).

A variety of stressors, including chemotherapeutic agents such as Triptolide (TPL) and endocrine disturbances like hyperandrogenism, can compromise mitochondrial membrane integrity in ovarian cells. Although mechanistic insights into mitochondrial membrane disruption and mtDNA release were initially derived from non-ovarian models, based on studies in other senescent or somatic cells, it is plausible that similar processes occur in aging ovarian cells. Disruption of mitochondrial membranes may facilitate the leakage of mitochondrial DNA (mtDNA) into the cytosol, where it is recognized by cGAS and triggers activation of the cGAS–STING pathway (McArthur et al., 2018; Cheng et al., 2025). Subsequent activation of this pathway amplifies inflammatory signaling through NF-κB and IRF3, leading to increased expression of pro-inflammatory cytokines and interferon-stimulated genes (ISGs). Under conditions of sustained activation, these inflammatory mediators not only exacerbate local inflammation but also impair mitochondrial respiratory chain function, potentially further enhancing ROS production (Rongvaux et al., 2014). In addition, NF-κB can modulate the transcription of mitochondrial enzymes and antioxidant systems, thereby contributing to ROS accumulation (Nisr et al., 2019), while IRF3/type I interferon signaling may indirectly increase ROS levels by altering mitochondrial membrane potential and bioenergetic balance (Nanayakkara et al., 2019; Abad et al., 2024).

Together, these events establish a self-reinforcing loop in which elevated ROS further damages mitochondrial membranes, promotes additional mtDNA leakage, and induces secondary nuclear DNA damage, leading to the formation of micronuclei or cytoplasmic chromatin fragments (CCFs) (Xi et al., 2025). In aged granulosa cells, these increases in ROS and DNA damage may reflect both a consequence of age-associated decline in repair and antioxidant defenses and a driver that accelerates cellular senescence and functional deterioration.These cytosolic nucleic acids could perpetuate cGAS–STING activation and sustain inflammatory signaling, which, although direct evidence in naturally aged granulosa cells is limited, is supported by experimental models in which mtDNA leakage activates cGAS–STING and amplifies NF-κB/IRF3 signaling, reinforcing oxidative stress and inflammatory pathways (Hong et al., 2024; Mittal et al., 2014).

The impact of this ROS–mtDNA–cGAS–STING loop varies among ovarian cell types, reflecting their distinct metabolic demands and stress sensitivities. Granulosa cells appear particularly vulnerable to ROS accumulation, rapidly exhibiting mitochondrial dysfunction, functional decline, and senescent phenotypes (Wen et al., 2024; Zhao et al., 2025). Oocytes are highly sensitive to both oxidative stress and inflammatory cytokines, which impair meiotic maturation and developmental competence (Tariq et al., 2025). Stromal cells can further amplify local damage through paracrine inflammatory signaling, thereby contributing to progressive deterioration of the follicular microenvironment (Rowley et al., 2020).

Preclinical studies provide supporting evidence for this model. In mouse ovaries exposed to TPL or hyperandrogenism, mtDNA leakage is markedly increased and accompanied by heightened activation of the cGAS–STING–NF-κB/IRF3 axis, elevated inflammatory markers, increased ROS levels, and compromised follicle quality (Cheng et al., 2025; Cai et al., 2025). *In vitro* studies further demonstrate that inhibiting mtDNA release or pharmacologically blocking STING signaling attenuates ROS accumulation, alleviates inflammation, and delays follicular apoptosis (Zhao et al., 2024). Recent work has identified additional molecular components that are likely involved in this network, including the NLRP3 inflammasome, mitochondrial channel proteins such as VDAC1, and antioxidant enzymes (e.g., SOD2 and GPX4), collectively forming a complex regulatory circuit that promotes ovarian aging (Navarro-Pando et al., 2021; Hu et al., 2025; Yan et al., 2022). Clinically, elevated levels of cell-free mitochondrial DNA (cf-mtDNA) detected in ovarian tissue and follicular fluid from POI patients likely reflect sustained mitochondrial distress and chronic innate immune activation, although direct evidence linking these mechanisms to cGAS–STING activation in human ovarian cells remains limited (Zhou et al., 2023).

In summary, ROS appear to function as both signaling mediators and drivers of damage in ovarian aging. Chronic activation of the ROS–mtDNA–cGAS–STING–inflammation feedback loop, initiated by mitochondrial dysfunction, may accelerate granulosa cell and oocyte decline, remodels the ovarian microenvironment, and ultimately contributes to depletion of the follicle reserve. While this pathway offers promising therapeutic targets for POI, its redundant and self-amplifying nature suggests that multi-node or combinatorial interventions may be required to effectively mitigate ovarian functional decline.

### 3.3 Chronic low-grade inflammation and SASP

Chronic low-grade inflammation is widely recognized as a hallmark pathological feature of ovarian aging. With progressive decline in ovarian tissue function, persistent activation of the DNA damage response (DDR) occurs, accompanied by sustained secretion of pro-inflammatory cytokines, such as interleukin-6 (IL-6), tumor necrosis factor-α (TNF-α), and interleukin-1β (IL-1β), as well as chemokines including CCL2/MCP-1 and CXCL8/IL-8. Together, these factors could establish a state of chronic inflammatory signaling within the local ovarian microenvironment (Shen et al., 2023; Zhu et al., 2025). Although typically mild in magnitude, this inflammation may accumulate over time, potentially disrupting cellular homeostasis, promoting immune cell recruitment, and facilitating stromal remodeling and fibrosis. Increasing evidence suggests that chronic low-grade inflammation not only reflects functional dysregulation of senescent cells but also provides a permissive foundation for the development and maintenance of the senescence-associated secretory phenotype (SASP) (Hense et al., 2024; Pan et al., 2025).

SASP constitutes a central molecular node linking cellular senescence to persistent inflammation. It encompasses a broad spectrum of bioactive factors secreted by senescent cells, including canonical pro-inflammatory cytokines (IL-6, TNF-α, IL-1β), extracellular matrix-remodeling enzymes (MMP-3, MMP-9), pro-fibrotic mediators (transforming growth factor-β, TGF-β), and immune-modulatory molecules. Through autocrine and paracrine actions, these components propagate inflammatory signaling, amplify tissue damage, and induce secondary senescence in neighboring cells (Zhu et al., 2021; Yue et al., 2022; Ye et al., 2024). Within ovarian tissue, granulosa cells appear to represent a major source of SASP, while stromal and infiltrating immune cells may respond to SASP cues, collectively forming a multicellular inflammation–senescence network that reinforces ovarian dysfunction (Yan et al., 2024; Zhao et al., 2020). Importantly, genotoxic stress and inflammatory signaling are not restricted to granulosa cells. Theca cells, stromal fibroblasts, endothelial cells, and infiltrating immune cells within the ovary are likewise exposed to oxidative and inflammatory insults, and based on studies in other senescent or somatic cells, it is plausible that they contribute to cGAS–STING activation and SASP propagation, thereby reinforcing tissue-wide inflammatory and fibrotic remodeling (Zhu et al., 2025; Isola et al., 2024).

Mechanistically, SASP production is governed by multiple interconnected signaling pathways. The NLRP3 inflammasome acts as a sensor in many cell types of intracellular danger signals, including cytosolic DNA fragments and extracellular ATP, leading to caspase-1 activation and subsequent maturation and release of IL-1β and IL-18, thereby potentially generating a potent pro-inflammatory amplification loop (Kelley et al., 2019; Fujimura et al., 2023). In parallel, opening of mitochondrial membrane channels such as voltage-dependent anion channel 1 (VDAC1) facilitates mitochondrial DNA (mtDNA) leakage into the cytosol, which can activate both inflammasome signaling and the cGAS–STING pathway (Kim et al., 2019). Sustained activation of downstream transcriptional programs, particularly NF-κB and p38 MAPK signaling, appears to drive high-level expression of SASP-associated genes, including IL-6, CXCL8, MMP-3, MMP-9, and TGF-β, thereby maintaining senescent cells in a chronic secretory state (Kim et al., 2019; Niklander et al., 2021). In addition, DDR signaling contributes to SASP induction via the ATM–NF-κB axis, while epigenetic and metabolic regulators such as BRD4 and mTORC1 support the long-term stability of SASP gene expression (Zhao et al., 2020; Herranz and Gil, 2018). In the context of ovarian aging, these pathways establish a self-perpetuating inflammatory–senescence environment characterized by chronic inflammation, SASP production, and cellular damage. While this environment may create conditions under which cytosolic DNA accumulates and cGAS–STING could be engaged, it remains unclear whether SASP itself directly triggers cGAS–STING activation in ovarian cells. Consistent with this model, animal studies and *in vitro* analyses using PCOS or follicular fluid–based systems suggest that cGAS–STING activation is associated with granulosa cell inflammation and apoptosis, whereas pharmacological or genetic inhibition of this pathway may attenuate inflammatory signaling and delays follicular dysfunction (Zhao et al., 2024; Ye et al., 2024). Additionally, cGAS–STING activation can influence the ovarian immune landscape by promoting recruitment of innate immune cells and shaping local inflammatory responses, further contributing to tissue senescence.

Beyond its effects on individual cells, SASP may exert broad paracrine influences that remodel the ovarian microenvironment, including alterations in vascular structure, extracellular matrix composition, and local immune status. These changes accelerate follicular aging, reduce the pool of developmentally competent follicles, and ultimately contribute to ovarian functional decline and the onset of POI (Hense et al., 2024; Yue et al., 2022; Isola et al., 2024). Collectively, these findings position SASP as a pivotal driver of ovarian aging through its ability to sustain and amplify chronic inflammation within a multicellular and multifactorial network. Nevertheless, most current evidence derives from in vitro–based models or short-term animal studies. The spatiotemporal dynamics of SASP secretion *in vivo*, as well as its precise impact across different stages of follicular development, remain incompletely understood and warrant further investigation using advanced tools such as SASP-reporter mouse models.

### 3.4 Apoptosis and autophagy imbalance

During ovarian aging, disruption of the balance between apoptosis and autophagy functions as a key mechanism driving follicular depletion and functional decline. Autophagy is a fundamental cellular homeostatic process responsible for the degradation of damaged mitochondria, misfolded protein complexes, and cytosolic DNA, thereby potentially preserving intracellular integrity and stress tolerance (Li et al., 2020). Under physiological conditions, granulosa cells and oocytes maintain moderate basal autophagy, which effectively clears stress-induced damage, limits accumulation of danger-associated molecular patterns, and supports follicle survival and maturation. However, with advancing age or exposure to metabolic or genotoxic stress, autophagic capacity in ovarian cells declines progressively, leading to the accumulation of damaged organelles, cytosolic DNA, and stress signals, thereby shifting the cellular fate toward apoptosis and establishing a maladaptive autophagy–apoptosis imbalance (Li et al., 2024; Cai et al., 2024; Dong et al., 2022).

Emerging evidence specifically in ovarian cells indicates that cGAS–STING signaling is engaged when cytosolic DNA accumulates due to impaired autophagy. For instance, granulosa cell–specific stress models, such as Immp2l deficiency, demonstrate that mitochondrial and cytosolic DNA accumulation activates cGAS–STING, triggers downstream NF-κB and interferon-stimulated gene expression, and promotes senescence and apoptosis; pharmacological inhibition of STING mitigates these effects (Pan et al., 2025). Similarly, in PCOS mouse models, hyperandrogenism-induced stress activates cGAS–STING–NF-κB signaling in granulosa cells, while STING inhibition reduces inflammatory cytokine expression and apoptosis, supporting a modulatory role of this pathway in ovarian cell stress responses (Cai et al., 2025; Zhao et al., 2024).

Mechanistically, in ovarian cells, reduced autophagic flux prevents efficient clearance of cytosolic DNA fragments, which then accumulate and engage cGAS, activating STING. Activated STING subsequently enhances NF-κB and p38 MAPK pathways, promoting the transcription of pro-inflammatory cytokines and apoptosis-related genes, intensifying Bax induction and Caspase-3 activation, and thereby accelerating apoptotic cell death (Li et al., 2022). Evidence in ovarian cells also suggests that cGAS–STING may participate in feedback regulation of autophagy, potentially engaging Beclin-1–dependent pathways, although the exact mechanisms in oocytes and granulosa cells remain to be fully elucidated (Gui et al., 2019; Lu et al., 2023).

Under conditions of sustained stress or age-associated autophagy impairment, persistent STING activation in ovarian cells can reinforce apoptotic and pro-inflammatory signaling. Preclinical studies using ovarian cell–specific models show that pharmacological inhibition of STING or enhancement of autophagy reduces cytosolic DNA accumulation, attenuates Caspase-3 activation, and mitigates apoptosis, supporting a modulatory role of cGAS–STING in the autophagy–apoptosis axis.

Collectively, these observations suggest that autophagy imbalance functions not only as an upstream trigger of apoptosis but also interacts with cGAS–STING signaling specifically in ovarian cells to amplify stress and senescence signals. Rather than acting in isolation, apoptosis and autophagy are tightly interconnected under complex regulatory networks, with cGAS–STING representing an influential, yet ovarian cell–specific, modulator. Future studies using ovarian cell–specific genetic models (e.g., granulosa-specific or oocyte-specific cGAS/STING knockout) are required to definitively establish the causal role of this pathway in age-related autophagy–apoptosis imbalance.

### 3.5 Other related mechanisms

Telomere shortening is a hallmark of cellular senescence, and telomere dysfunction can activate the DNA damage response (DDR), thereby serving as an initiating signal for cellular functional decline (Abdisalaam et al., 2020). Accumulating evidence suggests that telomere attrition may directly engage the cGAS–STING pathway through cytosolic DNA leakage, linking genomic instability to innate immune activation (Şerifoğlu et al., 2025). In a telomerase-deficient (tert^−/−^) zebrafish model, despite pronounced telomere shortening, genetic ablation of STING restored cellular proliferative capacity, reduced p53 expression, and decreased DNA damage markers such as γH2AX. These findings indicate that cGAS–STING functions as a critical signal amplifier in telomere dysfunction–induced senescence. Mechanistically, telomere attrition promotes the formation of micronuclei or chromosomal fragments that leak into the cytosol, where they are sensed by cGAS, leading to STING activation and induction of type I interferons and pro-inflammatory cytokines. This process establishes a chronic low-grade inflammatory milieu that accelerates tissue aging.

This mechanism has also been observed in human tissues (Şerifoğlu et al., 2025). For example, alveolar type II epithelial (ATII) cells from aged donors exhibit telomere shortening accompanied by accumulation of cytosolic double-stranded DNA and activation of the cGAS–STING pathway, as evidenced by IRF3 upregulation and increased pro-inflammatory cytokine expression. Together, these observations support the concept that telomere dysfunction promotes DNA leakage and cGAS–STING activation, thereby amplifying aging-associated inflammatory signals in experimental systems and correlating with chronic inflammation and cellular dysfunction in clinical contexts. These findings raise the possibility that telomere–cGAS–STING signaling may represent a potential intervention axis to delay ovarian aging (Ebata et al., 2022), although direct evidence in ovarian tissue remains limited.

Beyond telomere attrition, cells undergoing prolonged replication or entering senescence frequently exhibit extensive chromatin remodeling, altered histone modifications, and aberrant DNA methylation patterns. Such epigenetic alterations can substantially reshape the transcriptional programs governing DNA repair, cell cycle control, and immune responses (Joshi et al., 2017). Notably, in cGAS-deficient mouse embryonic fibroblasts (MEFs), telomere shortening and cell cycle arrest still occur; however, p21 protein expression and senescence-associated β-galactosidase positivity are reduced. These findings suggest that cGAS may not only function as a cytosolic DNA sensor but may also modulate the execution or reinforcement of telomere-driven senescence programs (Yang et al., 2017a).

In parallel, epigenetic regulators can directly influence DNA sensing capacity. For instance, the arginine methyltransferase PRMT5 methylates the DNA sensor IFI16, reducing its affinity for double-stranded DNA and thereby suppressing downstream activation of the cGAS–STING–TBK1–IRF3 signaling cascade. This modulation ultimately attenuates type I interferon production and pro-inflammatory cytokine expression (Kim et al., 2020; Dai et al., 2022). Collectively, these findings outline a mechanistic continuum in which epigenetic dysregulation alters DNA sensing thresholds, facilitating cGAS–STING activation and promoting cellular senescence.

Although direct experimental validation of these mechanisms in ovarian cells is still scarce, they have been consistently demonstrated across multiple *in vitro* systems and somatic tissue models. Given that granulosa cells, oocytes, and supporting ovarian somatic cells are exposed to prolonged replicative stress, oxidative damage, and age-associated epigenetic drift, telomere dysfunction and epigenetic aberrations are likely to intersect with cGAS–STING signaling during ovarian aging. Nevertheless, extrapolation from somatic cell models to the ovarian context—particularly to oocytes, which are terminally differentiated and non-renewable—requires caution. The unique telomere maintenance strategies and epigenetic landscape of oocytes may confer distinct regulatory features to cGAS–STING signaling that remain to be elucidated.

## 4 Intervention strategies and potential applications

In recent years, with increasing insights into the molecular mechanisms of ovarian aging and POI, the cGAS–STING pathway has emerged as a critical hub linking DNA damage, mitochondrial dysfunction, and chronic inflammation, making it a potential therapeutic target. Preclinical studies specifically in ovarian models have demonstrated that modulation of cGAS–STING signaling can improve follicular function and reduce ovarian cell stress (Pan et al., 2025; Zhao et al., 2024). Pharmacological strategies targeting this pathway and its upstream regulators can be broadly categorized into three main approaches: (i) direct inhibition of cGAS or STING activity to block inflammatory signaling; (ii) preservation of mitochondrial homeostasis to reduce cytosolic mtDNA leakage; and (iii) reduction of upstream stressors through antioxidants and enhancement of DNA repair. These strategies provide both theoretical and pharmacological bases for delaying ovarian cell senescence and preserving follicle reserve (see Table 1).

**TABLE 1:** Targeting the cGAS–STING pathway in ovarian aging and premature ovarian insufficiency.

Intervention type	Compound	Target	Mechanism of action	Ref./Notes
Direct inhibition	RU.521	cGAS	Competitively binds to the catalytic pocket, preventing DNA entry and inhibiting cGAMP synthesis	Vincent et al. (2017); preclinical, mouse models
	TDI-6570	cGAS	Occupies the catalytic pocket with improved selectivity, suppressing cGAMP generation	Mankan et al. (2014); preclinical, *in vitro* and *in vivo* inflammation models
	PF-06928215	cGAS	Occupies the catalytic pocket with improved selectivity, suppressing cGAMP generation	Cornelis et al. (2020); preclinical, *in vitro* and *in vivo*
	G150	cGAS	Reversibly inhibits both human and murine cGAS activity	Lama et al. (2019); preclinical, *in vitro* and *in vivo*, cross-species studies
	H-151	STING	Covalently modifies key cysteine residues, blocking STING translocation and IRF3 phosphorylation	Haag et al. (2018); preclinical, mouse and autoimmune models
	C-176/C-178	STING	Allosterically prevents STING dimerization, blocking downstream signal activation	Mukai et al. (2016); preclinical, autoimmune models
	SN-011	STING	Occupies the ligand-binding pocket, blocking signal transduction	Li et al. (2025); preclinical, inflammation models
	Astin C	STING–TBK1 complex	Inhibits STING–TBK1 interaction, reducing IRF3 activation	Li et al. (2018); preclinical, *in vitro*
Mitochondrial homeostasis modulators	MitoQ	Mitochondria	Mitochondria-targeted ROS scavenger that restores membrane potential and energy metabolism	Wang et al. (2023); preclinical, animal and *in vitro* studies
	SS-31 (Elamipretide)	Mitochondrial membrane	Stabilizes membrane potential and optimizes mitochondrial energy metabolism	Yao et al. (2024); preclinical, ovarian tissue culture and oocytes
	CoQ10	Electron transport chain	Improves electron transport efficiency and reduces ROS generation	Ben-Meir et al. (2015); clinical trials in assisted reproduction; animal studies
	NMN/NR	SIRT family/NAD^+^ metabolism	Increases NAD^+^ levels, activating SIRT-mediated autophagy and DNA repair pathways	Liang et al. (2020); preclinical, animal studies; emerging clinical studies in metabolism
	Spermidine	Mitophagy pathway	Promotes mitophagy to maintain cellular homeostasis	Zhang et al. (2023); preclinical, animal studies
Antioxidants	N-acetylcysteine (NAC)	Glutathione system	Enhances GSH levels to lower ROS and protect granulosa and oocyte cells	Yang et al. (2017b); preclinical, *in vitro*/animal models; some clinical evidence in oxidative stress conditions
	Resveratrol (RSV)	SIRT1/3 pathway	Activates SIRT1/3 signaling, improves mitochondrial function, reduces oxidative stress	Okamoto et al. (2022); preclinical and assisted reproduction clinical studies
	Melatonin (MT)	Free radical scavenging/mitochondrial membrane	Scavenges free radicals and stabilizes mitochondrial membrane potential	Tamura et al. (2020); preclinical and ART clinical trials
	Taurine, EGCG, Theaflavin, Vitamins E/C	Antioxidant/DNA protection systems	Reduce ROS and DNA damage, indirectly suppress cGAS–STING activation	Shang et al. (2024); preclinical/antioxidant studies; clinical data limited
DNA repair enhancers	NMN/NR	NAD^+^/PARP/SIRT1/6 pathways	Activate DNA repair networks, reducing double-strand breaks and cytosolic DNA accumulation	Huang et al. (2022); preclinical, animal studies
	Resveratrol (RSV)	SIRT1	Promotes DNA repair and anti-inflammatory responses, delaying cellular senescence	Wang et al. (2022); preclinical/some assisted reproduction trials
	EGCG/Riboflavin (Vitamin B_2_)	DNA repair enzymes	Enhance DNA repair capacity and reduce ROS-induced DNA breaks	Tobi et al. (2002), Dricot et al. (2024); preclinical
	ATM/ATR kinase modulators	DNA damage response	Regulate recognition and repair of double-strand breaks	Weber and Ryan (2015); preclinical, *in vitro*/animal models
Combined modulation	TLR2 agonist + STING activator	TLR2/STING	TLR2 agonist can reprogram downstream STING signaling, enhancing NF-κB activation while modulating IRF3 output	Song et al. (2025); preclinical, tumor models; not yet tested in ovarian aging

This table summarizes representative compounds targeting the cGAS–STING, pathway and upstream cellular stress responses relevant to ovarian aging.

Abbreviations: cGAS, cyclic GMP–AMP, synthase; STING, stimulator of interferon genes; TBK1, TANK-binding kinase 1; IRF3, interferon regulatory factor 3; ROS, reactive oxygen species; ETC, electron transport chain; NAD^+^, nicotinamide adenine dinucleotide; NMN, nicotinamide mononucleotide; NR, nicotinamide riboside; SIRT, sirtuin family; GSH, glutathione; EGCG, epigallocatechin gallate; ATM, ataxia telangiectasia mutated; ATR, ATM- and, Rad3-related kinase; TLR2, Toll-like receptor 2.

### 4.1 Direct inhibition of cGAS–STING signaling

The cGAS–STING pathway plays a pivotal role in ovarian aging and POI progression. Direct inhibition of this pathway has attracted considerable attention as a potential therapeutic strategy. Multiple small-molecule inhibitors targeting either cGAS or STING block inflammatory signaling and reduce ovarian cell senescence in preclinical models.

Activation of cGAS requires binding to cytosolic DNA, after which cGAS catalyzes the synthesis of the cyclic dinucleotide 2′3′-cGAMP, triggering downstream STING-mediated inflammatory responses. Consequently, inhibitors targeting cGAS generally operate via two mechanisms: blocking cGAS–DNA binding, or suppressing its catalytic activity, thereby reducing cGAMP production at the initiation stage of the pathway and attenuating type I interferon and pro-inflammatory cytokine release. Among these, RU.521 was the first structurally characterized small molecule to competitively bind cGAS, preventing DNA entry into the catalytic pocket and significantly reducing type I interferon levels in mouse models (Vincent et al., 2017). Subsequent compounds, TDI-6570 and PF-06928215, exhibit improved selectivity for the cGAS catalytic site, enhancing inhibition of cGAMP generation in both *in vitro* and *in vivo* inflammation models (Mankan et al., 2014; Cornelis et al., 2020). G150 has demonstrated reversible inhibition of both human and murine cGAS, providing a platform for cross-species studies (Lama et al., 2019). Inhibitors that disrupt cGAS–DNA condensate formation target the emerging mechanism of phase separation with high specificity. However, their efficacy and safety in ovarian aging models remain largely unexplored, and cell-specific delivery represents a major translational challenge.

STING, as the direct receptor of cGAMP, undergoes multi-step activation involving ligand binding, dimerization, and palmitoylation. STING inhibitors block downstream inflammatory signaling by interfering with these regulatory steps. For example, H-151 covalently modifies key residues of STING, preventing its translocation and inhibiting IRF3 phosphorylation, thereby mitigating systemic inflammation (Haag et al., 2018). C-176/C-178 act via an allosteric mechanism to prevent STING dimerization and show effective anti-inflammatory activity in autoimmune models (Mukai et al., 2016); SN-011 occupies the ligand-binding site of STING, demonstrating pronounced anti-inflammatory effects in preclinical models (Li et al., 2025), and the natural cyclic peptide Astin C inhibits the STING–TBK1 interaction, reducing IRF3 activation and inflammatory signaling (Li et al., 2018).

Although these small molecules have shown promising results in inflammation and aging models, their application in human ovarian aging and POI remains at an early exploratory stage. A critical limitation is that the cGAS–STING pathway also plays essential roles in antitumor immunity, and long-term systemic inhibition may carry potential risks, such as increased tumor susceptibility, which must be rigorously evaluated in future clinical translation efforts.

### 4.2 Upstream protective strategies

Mitochondrial dysfunction and oxidative stress are considered core drivers of ovarian aging. Declines in mitochondrial membrane potential, excessive ROS production, and mtDNA leakage not only directly damage granulosa and oocyte cells, but also activate the cGAS–STING pathway, inducing chronic inflammation and senescence-associated secretory phenotype (SASP). To target these pathological events, a series of upstream protective strategies have been developed, including mitochondria-targeted antioxidants, broad-spectrum antioxidants, and DNA repair enhancers, which can maintain cellular homeostasis and reduce ROS and mtDNA leakage, thereby indirectly suppressing aberrant cGAS–STING activation.

Mitochondria-targeted coenzyme Q10 (Mitoquinone, MitoQ) selectively accumulates within mitochondria to scavenge ROS, significantly restoring membrane potential and improving oocyte maturation and follicle viability (Wang et al., 2023). The mitochondrial protective peptide SS-31 (Elamipretide) stabilizes membrane potential and optimizes mitochondrial energy metabolism, enhancing mitochondrial homeostasis and developmental competence in thawed ovarian tissue and *in vitro* cultured oocytes (Yao et al., 2024). Coenzyme Q10 (CoQ10) improves electron transport chain efficiency, reducing ROS generation and ameliorating oocyte mitochondrial function, demonstrating anti-aging effects in animal studies and assisted reproduction clinical trials (Ben-Meir et al., 2015). Nicotinamide mononucleotide (NMN) and Nicotinamide riboside (NR) not only enhance mitochondrial metabolism but also improve follicle reserve and oocyte quality via sirtuin (SIRT)-mediated autophagy and DNA repair pathways (Liang et al., 2020); Spermidine promotes mitophagy to maintain cellular homeostasis, further improving oocyte function (Zhang et al., 2023). Collectively, restoring mitochondrial homeostasis not only optimizes ovarian bioenergetics but also reduces ROS and mtDNA leakage, providing a theoretical basis for indirect inhibition of cGAS–STING signaling.

N-acetylcysteine (NAC) enhances glutathione levels to lower ROS and protect granulosa and oocyte cells (Yang et al., 2017b). Resveratrol (RSV) activates SIRT1/3 pathways to improve mitochondrial function, reduce oxidative stress, and has been shown to enhance oocyte quality and follicle viability in mice and assisted reproduction populations (Okamoto et al., 2022). Melatonin (MT), as an endogenous antioxidant, directly scavenges free radicals and stabilizes mitochondrial membrane potential, improving oocyte quality and reducing ovarian oxidative stress in both animal experiments and human ART trials (Tamura et al., 2020). Other antioxidants, including Taurine, Epigallocatechin gallate (EGCG), Theaflavin (TF), and Vitamins E/C, also reduce ROS, alleviate DNA damage, and indirectly suppress cGAS–STING activation through anti-inflammatory effects (Shang et al., 2024). These agents can act synergistically with mitochondrial homeostasis modulators, collectively mitigating oxidative stress and inflammatory burden to delay ovarian aging.

NMN and NR increase intracellular NAD^+^ levels, activating DNA repair pathways including poly ADP-ribose polymerase (PARP) and SIRT1/6, thereby reducing double-strand breaks and cytosolic DNA accumulation and attenuating cGAS–STING activation (Huang et al., 2022). Resveratrol similarly promotes DNA repair via SIRT1 signaling, delaying cellular senescence (Wang et al., 2022). EGCG and Riboflavin (Vitamin B_2_) have been shown in basic studies to enhance DNA repair capacity and reduce ROS-induced DNA breaks (Tobi et al., 2002; Dricot et al., 2024). Although data in ovarian models are limited, small-molecule modulators of ATM and ATR kinases, established regulators of DNA damage response, have demonstrated potential for broader applications in DNA repair and warrant exploration in ovarian aging studies (Weber and Ryan, 2015).

Together, mitochondrial homeostasis modulators, antioxidants, and DNA repair enhancers form a multilayered intervention network, from upstream control of ROS and energy metabolism to DNA damage repair. This network not only improves ovarian cell function but also indirectly prevents excessive cGAS–STING activation, offering a pharmacological framework for delaying ovarian aging and premature ovarian insufficiency.

Given that ovarian aging is driven by upstream mitochondrial dysfunction, oxidative stress, and DNA damage, together with downstream overactivation of cGAS–STING signaling, combinatorial strategies targeting both upstream and downstream nodes may provide more effective protection than single-target interventions. For example, enhancing DNA repair capacity may reduce cytosolic DNA accumulation, while pharmacological inhibition of STING can directly suppress downstream inflammatory signaling, thereby alleviating chronic inflammation and the SASP phenotype from multiple levels.

Beyond direct inhibition, emerging studies in other disease contexts suggest that qualitative modulation of cGAS–STING downstream signaling outputs could provide a more refined therapeutic concept. In oncology models, persistent STING activation has been shown in certain settings to promote an immunosuppressive microenvironment; notably, co-administration of a TLR2 agonist was reported to “reprogram” STING downstream signaling by enhancing NF-κB activity while attenuating IRF3-dependent interferon responses, thereby overcoming therapeutic resistance (Song et al., 2025). Importantly, these observations are derived exclusively from tumor systems and should be regarded as a forward-looking hypothesis when extrapolated to ovarian biology, as direct experimental evidence supporting such signaling modulation in ovarian tissue is currently lacking. Moreover, the ovarian system—particularly the presence of non-renewable oocytes with highly specialized genomic and mitochondrial regulation—introduces unique biological risks and constraints, raising the possibility that interference with innate immune signaling outputs may have unintended consequences distinct from those observed in proliferative tumor cells. Nevertheless, despite these limitations, this oncology-informed framework provides a conceptual basis for cautiously exploring whether selective downstream signaling modulation, rather than global pathway inhibition, could theoretically attenuate chronic inflammation while permitting adaptive tissue responses in ovarian aging models.

At present, robust clinical efficacy data directly targeting the cGAS–STING pathway in premature ovarian insufficiency are lacking. Most available evidence is derived from mechanistically informed preclinical studies or indirect clinical observations. Across diverse ovarian injury and aging models, interventions that attenuate aberrant cGAS–STING activation—either through direct pathway inhibition or upstream preservation of mitochondrial and genomic integrity—have been associated with functional improvements, supporting a mechanism–function alignment and suggesting translational relevance, even though definitive therapeutic efficacy in humans remains to be established.

## 5 Conclusion

The cGAS-STING signaling pathway emerges from this synthesis as a pivotal integrator of damage signals and a central driver of the sterile inflammatory microenvironment that characterizes ovarian aging. This review has delineated the mechanisms by which cGAS-STING activation links upstream insults—such as nuclear DNA damage, mitochondrial dysfunction, and oxidative stress—to downstream pathological outcomes, including cellular senescence, apoptosis, and the profound remodeling of the follicular microenvironment through the SASP. This positioning underscores its potential as a theoretical linchpin explaining the accelerated and often irreversible nature of functional decline in both physiological ovarian aging and its pathological acceleration in POI.

A critical insight from this analysis is the likely cell-type-specific nature of cGAS-STING signaling within the heterogeneous architecture of the ovary. Current evidence, largely derived from in vitroand animal models, suggests that granulosa and stromal cells are highly responsive to DNA damage and exhibit pronounced pathway activation, whereas oocytes may act as more passive recipients of inflammatory signals. This heterogeneity implies that the functional consequences of pathway activation are not uniform, raising key unanswered questions about differential activation thresholds, signaling outputs, and net effects on follicle survival versus atresia across distinct ovarian cell populations. Elucidating this specificity is paramount, not only for a fundamental understanding of ovarian biology but also for the development of targeted therapeutic strategies that minimize off-target effects on essential reproductive and systemic immune functions.

Furthermore, the data compellingly support the existence of a self-reinforcing, positive feedback loop involving cGAS-STING, mitochondrial distress, and oxidative stress. This vicious cycle, wherein ROS and DNA damage trigger cGAS-STING activation, which in turn exacerbates mitochondrial dysfunction and ROS production, creates a powerful engine driving progressive ovarian functional decline. The implication is that once a critical threshold of damage is surpassed, the system may be propelled toward failure in a manner that is difficult to halt or reverse through endogenous repair mechanisms alone. This loop provides a mechanistic explanation for the relentless progression observed in ovarian aging and underscores why interventions targeting a single node might be therapeutically limited, pointing instead to the potential superiority of combinatorial approaches. Importantly, although this review does not aim to provide a formal evaluation of therapeutic efficacy, the mechanistic framework outlined here offers a rationale for understanding why certain interventions demonstrate functional benefits in experimental and early translational settings. Suppression of chronic inflammatory signaling, mitigation of oxidative stress, and preservation of mitochondrial integrity—hallmarks of effective cGAS–STING modulation—have repeatedly been associated with improved follicular survival, stabilization of ovarian reserve markers, and enhanced cellular resilience. These observations suggest that mechanistic targeting of the cGAS–STING pathway may yield measurable functional advantages, even if long-term clinical outcomes require further validation.

From a translational perspective, targeting the cGAS-STING pathway presents a promising yet complex frontier. Pharmacological strategies can be broadly categorized into direct inhibition of the pathway’s core components (cGAS or STING) and upstream interventions aimed at preserving mitochondrial and genomic homeostasis to prevent aberrant activation. While preclinical studies using small-molecule inhibitors are encouraging, the dual role of cGAS-STING in tumor surveillance poses a significant challenge for long-term systemic inhibition. Therefore, future success will likely depend on developing ovary-specific delivery systems (e.g., leveraging nanocarriers or viral vectors) and exploring novel concepts such as “signal reprogramming”—modulating the downstream output of STING to favor protective over detrimental responses. This approach aims to achieve a delicate balance: suppressing the chronic inflammatory drive of ovarian aging without compromising systemic innate immunity.

Several promising future research directions emerge from this analysis. First, there is an urgent need to characterize the dynamics of cGAS-STING signaling in human ovarian tissue across the aging spectrum, utilizing tools like single-cell and spatial transcriptomics to map its activity with cellular resolution. Second, the role of negative feedback mechanisms, such as the local degradation of cGAMP by enzymes like ENPP1, in ovarian cells remains entirely unexplored; their dysfunction could be a key factor in sustaining pathway activation. Third, the development of reliable biomarkers of cGAS-STING activity (e.g., cf-mtDNA, cGAMP in follicular fluid) is essential for stratifying patients and monitoring therapeutic responses in future clinical trials. Finally, the exploration of combination therapies that simultaneously target upstream inducers (e.g., with antioxidants) and downstream effectors (e.g., with SASP modulators) represents a logical and potentially more effective strategy than monotherapies.

In conclusion, the cGAS-STING pathway is established as a critical mechanism linking cellular damage to sterile inflammation in the aging ovary. While significant progress has been made in understanding its basic role, substantial gaps remain, particularly regarding its cell-specific functions in humans, the safety of long-term modulation, and the feasibility of targeted interventions. Bridging these gaps will require a concerted effort integrating fundamental mechanistic research with innovative translational approaches. The ultimate goal is to translate these insights into strategies that can preserve ovarian function, not merely for extending fertility but for promoting the long-term cardiometabolic, skeletal, and cognitive health of women.

### 5.1 Permission to reuse and copyright

The figures in this manuscript were created by the authors using a licensed scientific illustration platform. Usage permissions were obtained with the authorization codes ARWTYbdbbd and WYPIW98458.

## Glossary

2′3′-cGAMP/cGAMP: Cyclic GMP–AMP (2′3′-cyclic GMP–AMP)
AMP: Adenosine monophosphate
ATII: Alveolar type II epithelial cells
ATP: Adenosine triphosphate
Bax: Bcl-2-associated X protein (pro-apoptotic factor)
Beclin-1: Autophagy protein Beclin-1
BRD4: Bromodomain containing protein 4
CCF: Cytoplasmic chromatin fragment
cGAS: Cyclic GMP–AMP synthase
cf-mtDNA: Cell-free mitochondrial DNA
CCL2/MCP-1: C-C motif chemokine ligand 2/Monocyte chemoattractant protein-1
CXCL8/IL-8: C-X-C motif chemokine ligand 8/Interleukin-8
DDR: DNA damage response
DNA: Deoxyribonucleic acid
dsDNA: Double-stranded DNA
EGCG: Epigallocatechin gallate
ER: Endoplasmic reticulum
ERGIC: ER–Golgi intermediate compartment
ENPP1: Ectonucleotide pyrophosphatase/phosphodiesterase 1
GMP: Guanosine monophosphate
GTP: Guanosine triphosphate
GPX4: Glutathione peroxidase 4
**H**_**2**_ **O**_**2**_ **(H2O2)**: Hydrogen peroxide
HRT: Hormone replacement therapy
IFI16: Interferon gamma inducible protein 16
IFN/IFN-α/β: Interferon/Interferon-alpha/Interferon-beta
IL-1β: Interleukin-1 beta
IL-6: Interleukin-6
IRF3: Interferon regulatory factor 3
ISG/ISGs: Interferon-stimulated gene(s)
LC3: Microtubule-associated protein 1 light chain 3
LIR: LC3-interacting region
MEFs: Mouse embryonic fibroblasts
MitoQ: Mitoquinone
MMP-3/MMP-9: Matrix metalloproteinase-3/Matrix metalloproteinase-9
MT/Melatonin: Melatonin
NMN: Nicotinamide mononucleotide
NLRP3: NOD-like receptor family pyrin domain containing 3
**NAD** ^**+**^ **(NAD+)**: Nicotinamide adenine dinucleotide (oxidized form)
NAC: N-acetylcysteine
NF-κB: Nuclear factor kappa-light-chain-enhancer of activated B cells
NR: Nicotinamide riboside
**O**_**2**_^**−**^ **(O2−)**: Superoxide anion
PARP/PARP1: Poly(ADP-ribose) polymerase
PCOS: Polycystic ovary syndrome
p21: Cyclin-dependent kinase inhibitor 1 (CDKN1A)
PRMT5: Protein arginine methyltransferase 5
POI: Premature ovarian insufficiency
p38 MAPK: p38 mitogen-activated protein kinase
ROS: Reactive oxygen species
RSV: Resveratrol
Rubicon: Protein name (kept as original)
SASP: Senescence-associated secretory phenotype
SIRT/SIRT1/SIRT3/SIRT6: Sirtuin family proteins
SOD2: Superoxide dismutase 2
SS-31 (Elamipretide): Mitochondrial protective peptide
STING: Stimulator of interferon genes
TBK1: TANK-binding kinase 1
TGF-β1: Transforming growth factor beta-1
TF (Theaflavin): Theaflavin (tea polyphenol)
TNF-α: Tumor necrosis factor-alpha
TPL: Triptolide
TREX1: Three prime repair exonuclease 1
VDAC1: Voltage-dependent anion channel 1
OH (hydroxyl radical): Hydroxyl radical
